# Elevated systemic levels of the matrix metalloproteinase inhibitor TIMP-1 correlate with clinical markers of cachexia in patients with chronic pancreatitis and pancreatic cancer

**DOI:** 10.1186/s12885-018-4055-9

**Published:** 2018-02-02

**Authors:** Olga Prokopchuk, Barbara Grünwald, Ulrich Nitsche, Carsten Jäger, Oleksii L. Prokopchuk, Elaine C. Schubert, Helmut Friess, Marc E. Martignoni, Achim Krüger

**Affiliations:** 1 Klinik und Poliklinik für Chirurgie, Klinikum rechts der Isar, Technische Universität München, Ismaninger Str. 22, 81675 Munich, Germany; 2 Institut für Molekulare Immunologie und Experimentelle Onkologie, Technische Universität München, Munich, Germany; 3 Medizinische Klinik II, Klinikum Obergöltzsch, Rodewisch, Germany; 4 Institut für Radiology, Klinikum rechts der Isar, Technische Universität München, Munich, Germany

**Keywords:** TIMP-1, Cachexia biomarker, Pancreas, Jaundice

## Abstract

**Background:**

Tissue inhibitor of metalloproteinases-1 (TIMP-1) is a candidate diagnostic and prognostic biomarker for pancreatic ductal adenocarcinoma (PDAC). Here, we determined the possible association of systemic TIMP-1 levels with cachexia and jaundice, two common PDAC-associated conditions.

**Methods:**

Plasma TIMP-1 was measured by ELISA in patients diagnosed with PDAC (*n* = 36) and chronic pancreatitis (CP) (*n* = 25). Patients without pancreatic pathologies and known malignancies of other origin served as controls (*n* = 13). TIMP-1 levels in these patients were tested for asscociation with jaundice and chachexia, and furthermore correlated with cachexia-related clinical parameters such as weight loss and ferritin, parameters of lung function, hemoglobin and liver synthesis parameters.

**Results:**

TIMP-1 plasma levels were mostly higher in CP and PDAC patients with concomitant jaundice or cachexia. Elevated plasma TIMP-1 levels were also associated with clinical cachexia markers, including absolute and relative values of weight loss and lung function, as well as ferritin, hemoglobin, and cholinesterase levels. TIMP-1 levels significantly correlated with cachexia only in patients without jaundice. Jaundice also impaired the use of TIMP-1 as a prognostic marker in cancer patients. Relating to cachexia status alone, a slightly improved association of TIMP-1 levels with survival of PDAC patients was observed.

**Conclusion:**

This retrospective study reports for the first time that plasma levels of TIMP-1 are associated with pancreatic lesion-induced cachexia in patients without jaundice. TIMP-1 is counterindicated as a survival marker in patients with jaundice.

## Background

Tissue inhibitor of metalloproteinases-1 (TIMP-1) correlates with tumor progression [1–5], and elevated levels of TIMP-1 in tumor tissue and in peripheral blood are associated with poor clinical outcome in numerous malignancies, including colorectal cancer [1, 2, 6, 7], breast cancer [3, 5], gastric cancer [8, 9], non-small cell lung cancer [10], and esophageal cancer [4]. TIMP-1 is increasingly recognized as a molecule with a variety of pro-tumorigenic functions, e.g. TIMP-1 can bind to the tetraspanin CD63 and promote liver metastases via both, host-mediated mechanisms as well as direct effects on tumor cell aggressiveness [1, 11–13]. In specific, TIMP1 signaling via CD63 leads to activation of hepatic stellate cells, which create a pre-metastatic niche in the liver allowing efficient metastasis to this organ [14], and also induces a tumor-promoting stress response in tumor cells [11].

Several independent studies have reported TIMP-1 to be of prognostic and diagnostic value for pancreatic ductal adenocarcinoma (PDAC) [15, 16]. TIMP-1 transcripts are found in both stroma and tumor cells of human PDAC [17]. TIMP-1 expression positively correlates with the degree of desmoplasia in the tumor stroma [17], as well as with de-differentiation of pancreatic tumor cells [18]. In experimental mouse models of chronic pancreatitis (CP), TIMP-1 mRNA expression in the pancreas increases with disease progression, suggesting an important role of TIMP-1 in pancreatic fibrosis [19]. Expression of TIMP-1 is not only increased in early pancreatic lesions such as CP but also in pancreatic intra-epithelial neoplasia and late PDAC [14]. Importantly, plasma levels of TIMP-1 protein were found to be significantly elevated in PDAC and CP patients in an unbiased system-wide proteomics approach [20, 21], which further supports its potential usefulness as a diagnostic tumor marker.

PDAC is often associated with jaundice, and it was shown that TIMP-1 levels are significantly elevated in patients with PDAC-associated jaundice as well as with jaundice due to non-malignant conditions such as gallstones [22]. Cachexia is another common condition associated with progression of pancreatic lesions, occurring in almost 40% of patients with CP and PDAC, and is a strong prognostic factor for PDAC patients [23]. Association of TIMP-1 with cachexia was observed in several established animal cancer cachexia models including the rat hepatoma cancer cachexia model [24], Wistar rats bearing subcutaneous Walker256 carcinomas [25], and CD2F1 mice bearing C26 adenocarcinomas [26]. These data suggest that PDAC-associated conditions may influence systemic TIMP-1 levels, potentially through fibrotic remodeling and inflammation. As such, these conditions may either benefit or hamper the clinical usefulness of TIMP-1 as a biomarker. So far, there is no consensus whether PDAC patients with concomitant jaundice have to be excluded from use of TIMP-1 as a prognostic marker for pancreatic cancer patients. Further, the impact of cachexia on the usefulness of TIMP-1 as clinical progression and survival marker has not yet been evaluated. It is also not clear whether TIMP-1 alone or in combination with cachexia and jaundice may have improved prognostic value in PDAC.

In this retrospective study, we explore the impact of cachexia and jaundice on the diagnostic and prognostic value of TIMP-1, in CP and PDAC. We further determine the usefulness of plasma TIMP-1 levels as a cachexia biomarker. We report that TIMP-1 was associated with clinical markers of cachexia and with presence of cachexia in our cohort. While TIMP-1 was counterindicated as a marker in combination with jaundice, combining TIMP-1 with cachexia represents a promising combination of prognostic parameters.

## Methods

### Patients and tissue biopsies

This study was approved by the Ethics Committee of the Medical Faculty of the Technical University of Munich (Germany; #1946/07), and written consent was obtained from all participants before surgery or before blood sampling.

### Clinical parameters assessment

The analysis was conducted on a pseudonymized data set. The study population comprised patients suspicious for pancreatic cancer or chronic pancreatitis between 2008 and 2015 in the Department of Surgery, Klinikum Rechts der Isar, Munich, who agreed to participate in the study. Plasma samples were taken after written informed consent. The diagnosis was verified by postoperative definitive histological examination, or, in patients without surgery, by cytology or clinical/radiological information, to the best of our knowledge. Weight was measured at the time of admission to the hospital. Height and weight histories over the six months preceeding admission were collected by OP and OLP. Jaundice was defined as a serum total bilirubin level ≥ 2 mg/dl. Cachexia was defined as loss of more than 10% of the original body weight within the last six months before scheduled surgery. We modified the International Classification Framework definition of cachexia proposed by Fearon and co-workers [27] and extended the cut-off point for cachexia to 10% as described previously [23, 28–30], to unambiguously distinguish between patients with and without cachexia. This criterion was demonstrated to influence survival and performance status in PDAC patients [31, 32]. Patients without tumor diagnosis and history of chronic pancreatitis served as controls. The control samples were obtained from patients attending the same hospital. Spirometry provided a measurement of the lung function, as determined by forced vital capacity (FVC) and the forced expiratory volume in 1 s **(**FEV_1_), and was conducted as previously described [32].

### Laboratory examinations

Blood samples were analyzed at the Institute of Clinical Chemistry and Pathobiochemistry, Klinikum rechts der Isar, Munich, according to standard operating procedures. Blood was collected in a 9 ml EDTA tube, one 2.9 ml coagulation tube and one 9 ml serum tube (S-Monovette, Sarstedt, Nümbrecht, Germany), and mixed immediately by gently inverting the tube. Hemoglobin levels were determined by the sodium lauryl sulfate hemoglobin detection method, and leucocytes counts were determined by flow cytometry with integrated hydrodynamic focusing, using Sysmex XE 5000–2 or Sysmex XT 2000i hematology analyzers (Norderstedt, Germany). A photometric diazonium–based test was used to measure serum bilirubin. A photometric biuret–based test was used for measurement of serum protein and the bromcresol green reaction test was used to determine serum albumin. Serum concentrations of CRP were measured with an immunoturbidimetric assay. Cancer antigen 19–9 (CA 19–9) was evaluated by an electrochemiluminescence immunoassay. The measurements of bilirubin, protein, albumin, CRP and CA 19–9 (e 602 module) were performed on a Cobas 8000 platform (Roche Diagnostics, Mannheim, Germany).

### Measurement of fat and muscle tissue on computed tomography scans

Measurement of fat and muscle tissue on computed tomography scans was performed as described previously [33]. Patients received a contrast-enhanced computed tomography (CT) scan for initial cancer staging or validation of CP or routine diagnostic purposes. We quantified skeletal muscle and fat thicknesses, as well as *M. psoas* cross-sectional area. A venous phase of a CT scan of the abdomen was chosen. Six different values were taken, as previously described [32]: thickness of the perirenal fat, thickness of the medial subcutaneous fat; thickness of the lateral subcutaneous fat; thickness of muscles measured at two different locations (*musculus erector spinae* and *musculus psoas*), and *musculus psoas* area (measured as described previously [32]).

### ELISA

Blood samples were collected, and plasma was obtained within 30 min by centrifugation of whole blood for 15 min at 1000 g. Plasma samples were immediately snap-frozen in liquid nitrogen and stored at − 80 °C. TIMP-1 levels in plasma were determined using the DuoSet ELISA kit (R&D Systems) according to the manufacturer’s instructions. Each sample was analyzed in triplicate. Results of the ELISA were analyzed using ReaderFit.

### Statistical analyses

Statistical analysis was performed using the statistical software SPSS version 23.0 (Chicago, IL, USA). Associations between quantitative variables were tested by Spearman correlations. Normal distribution was tested by Shapiro-Wilk tests and visual inspection of the histograms. Groups were compared using Student’s t-test for independent samples in the case of normal distribution, or nonparametric Mann-Whitney test for independent variables in the absence of normal distribution. To derive optimal cut-off values of plasma TIMP-1 levels, maximally selected log-rank statistics performed by R Software version 2.13.0 (R Foundation for Statistical Computing, Vienna, Austria) were used. Additionally, the R-function *maxstat.test* was employed [34]. Time-dependent survival probabilities were estimated with the Kaplan-Meier method, and the log-rank test was used to compare independent subgroups.

## Results

### Patient cohort

Plasma samples from 74 patients (PDAC, *n* = 36; CP, *n* = 25, healthy controls, *n* = 13) were analyzed. PDAC and control patients were over 60 years old, while the median age of CP patients was 14 years less than of the control patients (*p* = 0.527), and 19 years younger than PDAC patients (*p* < 0.001). Of these, 13 PDAC patients (36%) and 6 CP patients (24%) were classified as cachectic, as defined in the methods section. Most of the PDCA patients without cachexia (70%) presented with the International Union Against Cancer (UICC) tumor stage 2 (defined as T3, N0, M0 or T1–3, N1, M0 [34, 35]). PDAC patients with cachexia presented mostly with UICC stage 4 (54%; defined as any T, any N, M1 [35, 36]). Of the 36 PDAC patients, 10 (28%) presented with jaundice. There was no significant difference in the distribution of patients according to American Society of Anesthesiologists (ASA) physical status stages (Table 1).

**Table 1:** Clinical parameters of patients

	Control	PDAC			CP		
		non-cachexia	cachexia	*p*	non-cachexia	cachexia	*p*
N	13	23	13		19	6	ns
Female	6 (46%)	11 (48%)	6 (46%)	ns	10 (53%)	2 (33%)	
Male	7 (54%)	12 (52%)	7 (54%)		9 (47%)	4 (67%	
Age, years	63 (19–78)	68 (41–88)	61 (52–77)	ns	50 (36–77)	49 (44–73)	ns
UICC stage	nr	I 2 (9%)		ns	nr	nr	
		II 16 (70%)	II 5 (38%)				
		III 2 (9%)	III 1 (8%)				
		IV 3 (12%)	IV 7 (54%)				
ASA I	18%	7%	9%	ns	23%		ns
II	73%	64%	64%		46%	67%	
III	9%	29%	27%		31%	33%	
Jaundice	0 (0%)	5 (22%)	5 (38%)	ns	0 (0%)	1 (17%)	ns
Weight loss, kg	0.0 (0)	2 (0–8)	12,5 (6–34)	< 0.001#	0 (0–6)	12,5 (8–40)	< 0.001##
Weight loss, %	0.0 (0)	3.0 (0–9.2)	14.0 (10.0–26.6)	< 0.001#	0 (0–9.1)	15.7 (11.1–28.6)	< 0.001##
Perirenal fat, mm	15.2 (6.7–32.5)	11.0 (2.0–39.0)	11.6 (4.0–26.2)	ns	6 (2.5–22.4)	7.0 (5.2–19.1)	0.013§§
M. erector spinae, mm	36.2 (28.9–39.8)	30.3 (18.7–39.1)	31.1 (26.0–41.4)	ns	32.1 (24.3–47.4)	40.5 (36.7–45.1)	0.019##
M. psoas, mm	37.0 (30.6–53.8)	38.0 (25.1–43.5)	32.8 (25.0–39.5)	ns	37.9 (20.7–51.2)	28.8 (22.6–35.7)	0.030°°
							0.039##
M. psoas area, mm^2^	1044,2 (876,2–1997,0)	1022,1 (608,7–1371,8)	702,1 (527,3–1075,2)	0.035°	943,5 (345,7–1971,5)	714,4 (565,9–931,3)	
				0.031#			
Subcutaneous fat medial, mm	17.8 (6.7–62.6)	14.0 (3.0–60.6)	20.7 (1.1–50.4)	ns	8.2 (0.6–62.3)	6.3 (2.5–31.2)	
Subcutaneous fat lateral, mm	45.0 (29.1–92.0)	37.1 (14.9–77.5)	38.3 (7.7–72.4)	ns	33.5 (4.3–94.5)	34.6 (22.3–45.3)	

Values are demonstrated as median (minimum-maximum)

ns not significant; nr not relevant

° PDAC with cachexia versus control; # PDAC without cachexia versus PDAC with cachexia

§§ CP without cachexia versus control; °° CP with cachexia versus control; ## CP without cachexia versus CP with cachexia

**Fig. 1:**
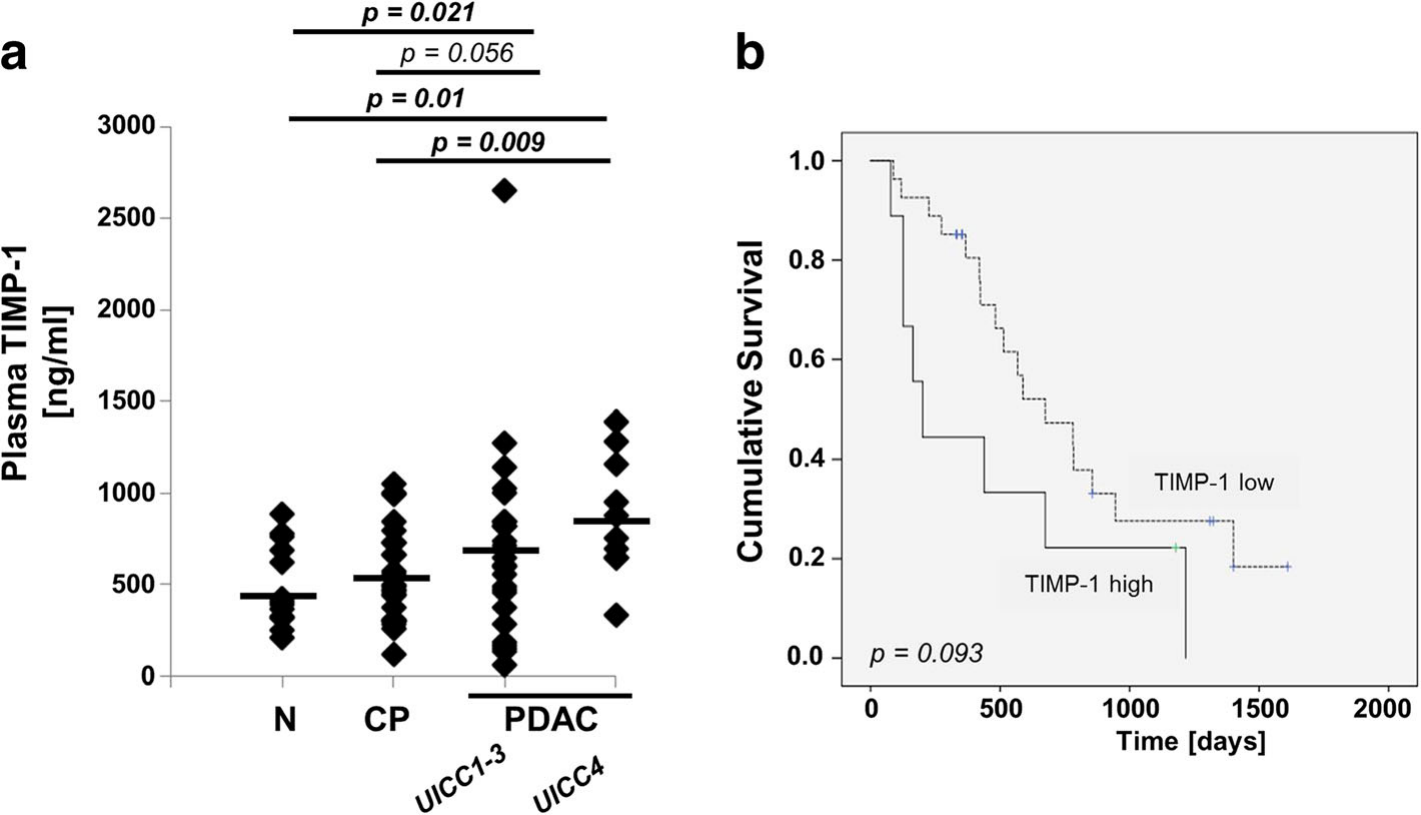
**a** Dot plots for plasma levels of TIMP-1 protein analyzed by ELISA in healthy individuals (control, *n* = 13), CP patients (*n* = 25), PDAC patients (UICC 1–3 (*n* = 26), and UICC 4 (*n* = 10)). Shapiro-Wilks testing in combination with visual inspection of the histograms showed that samples were not normally distributed. Groups were compared using nonparametric Mann-Whitney U test for independent variables. **b** Relation of plasma TIMP-1 to Kaplan Meier Overall Survival (OS) curves in PDAC patients. The optimal cut-off value of plasma TIMP-1 levels at 842 ng/ml was determined by maximally selected log-rank statistics. To consider multiple test issues within these analyses, the R-function *maxstat.test* was employed. For the cut-off at 842 ng/ml the Kaplan Meier curves were generated. Median OS for patients with low plasma TIMP-1 levels (*n* = 26): 453 days [440–768] 95% CI. Median OS for patients with high plasma TIMP-1 levels (*n* = 9): 202 days [117–817] 95% CI

TIMP-1 was previously shown to be of prognostic and diagnostic value in PDAC [15, 16]). First, we tested this observation in our patient cohort and confirmed that plasma TIMP-1 levels were significantly higher in PDAC patients compared to healthy controls and CP. This was true when comparing all PDAC (*p* = 0.021; *p* = 0.056) as well as PDAC patients with UICC stage 4 (*p* = 0.01; *p* = 0.009) (Fig. 1a). TIMP-1 significantly correlates with the presence of liver metastases (Table 2). The cut-off value of plasma TIMP-1 concentrations was defined as 842 ng/ml using maximally selected log-rank statistics performed by R Software. In PDAC patients, nine out of 36 (25%) had higher plasma TIMP-1 levels than the cut off value. The median overall survival (OS) was significantly higher in patients with low plasma TIMP-1 levels (*n* = 26, 453 days [440–768] 95% CI] as compared to patients with high plasma TIMP-1 levels (*n* = 9, 202 days [117–817, 95% CI] (*p* < 0.001). The Kaplan Meier analysis did not reach statistical significance in our cohort (*p* = 0.093, Kaplan Meier, log-rank test) (Fig. 1b).

**Table 2:** Correlations between plasma TIMP-1 and parameters of tumor load in pancreatic cancer patients

		T (1–4)	N (0,1)	Number of positive lymph nodes	Number of removed lymph nodes	Liver metastases (yes or no)	Distant metastases (yes or no)	UICC (1–4)	Grading (1–4)	Resection status (R0, R1)
Plasma TIMP-1, ng/ml	Spearman’s correlation	.138	−.061	−.189	.200	.375	.160	.222	−.026	.049
	Significance (2-tailed)	.491	.768	.355	.326	.024	.381	.194	.887	.821
	N	27	26	26	26	36	32	36	32	24

### TIMP-1 levels in patients with jaundice and cachexia

**Fig. 2:**
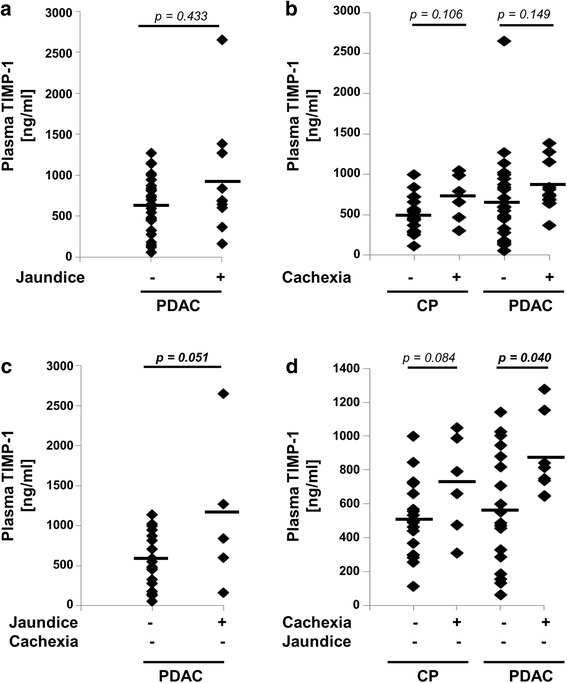
**a** Analysis of TIMP-1 in plasma samples in PDAC with (*n* = 10) and without jaundice (*n* = 26). Shapiro-Wilks testing in combination with visual inspection of the histograms showed that samples were not normally distributed. Groups were compared using nonparametric Mann-Whitney U test for independent variables. **b** Analysis of TIMP-1 in plasma samples in CP patients with (*n* = 6) and without cachexia (*n* = 19), and in PDAC patients with (*n* = 13) and without cachexia (*n* = 23). Shapiro-Wilks testing in combination with visual inspection of the histograms showed that samples were not normally distributed. Groups were compared using nonparametric Mann-Whitney U test for independent variables. **c** Analysis of TIMP-1 in plasma samples in PDAC with (*n* = 5) and without jaundice (*n* = 18) (patients with cachexia were excluded). Shapiro-Wilks testing in combination with visual inspection of the histograms showed that samples were normally distributed. Groups were compared using Student’s t-test for independent samples. **d** Analysis of TIMP-1 in plasma samples in CP patients with (*n* = 6) and without cachexia (*n* = 18), and in PDAC patients with (*n* = 8) and without cachexia (*n* = 18) (patients with jaundice were excluded). Shapiro-Wilks testing in combination with visual inspection of the histograms showed that samples were normally distributed. Groups were compared using Student’s t-test for independent samples

PDAC-related conditions can influence TIMP-1 levels secondary to PDAC and could therefore interfere with the use of TIMP-1 as a biomarker. Consistent with previous reports, [22] we confirmed mostly higher plasma TIMP-1 levels in PDAC patients with jaundice (*n* = 10) compared to patients without jaundice (*n* = 26) in our cohort (Fig. 2a). We also observed increased TIMP-1 plasma levels in CP and in PDAC patients with cachexia compared to those without, although these differences were not statistically significant (*p* = 0.149) (Fig. 2b). We then separated the effects of jaundice and cachexia within our cohort to test their individual associations with TIMP-1 levels. In non-cachectic patients, TIMP-1 plasma levels were significantly associated with jaundice (Fig. 2c). Furthermore, exclusion of jaundice patients revealed a clear association of TIMP-1 plasma levels with cachexia in CP and PDAC patients (Fig. 2d). Thus, plasma TIMP-1 levels are elevated in both, cachexia and jaundice patients due to the individual condition.

### TIMP-1 and clinical parameters of cancer cachexia

**Fig. 3:**
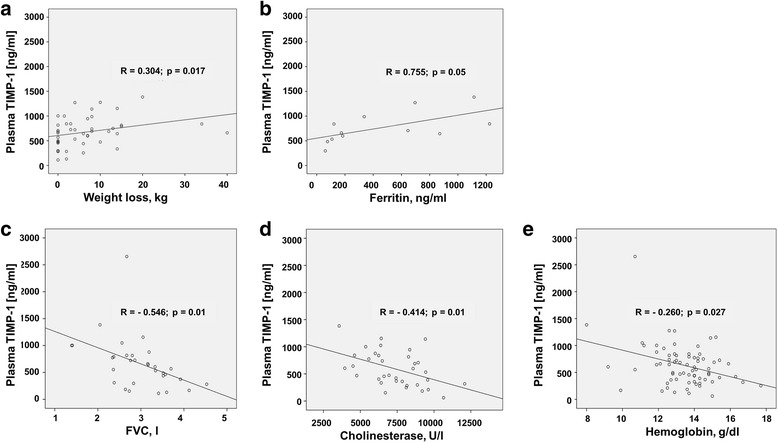
Associations between plasma TIMP-1 and weight loss (**a**), ferritin (**b**), FVC (**c**) cholinesterase (**d**) and hemoglobin (**e**) were tested by Spearman correlations

Next, we tested whether elevated TIMP-1 levels in cachexia may suggest a potential use as cachexia biomarker. We assessed clinical markers of cachexia and presence of cachexia in our patient cohort. There was a significant difference between the absolute and relative weight loss within the six months before diagnosis when comparing the cachexia and the non-cachexia PDAC group (*p* < 0.001). The mean value (± standard deviation) for the absolute weight loss was 2.3 ± 2.7 kg for non-cachexia patients and 13.7 ± 7.4 kg for cachexia patients. The mean value for the relative weight loss was 3.0 ± 3.5% for non-cachexia patients and 16.0 ± 6.4% for cachexia patients. The mean OS in patients with cachexia was lower, albeit not statistically significant (480 ± 68 versus 621 ± 104 days, *p* = 0.801). The *M. psoas* cross-sectional area was significantly lower in PDAC patients with cachexia as compared to controls and PDAC patients without cachexia (*p* = 0.035 and *p* = 0.031, respectively) (Table 1). The thickness of *M. erector spinae* and *M. psoas* was reduced in CP patients with cachexia as compared to CP patients without cachexia (Table 1). Elevated plasma TIMP-1 levels were significantly correlated with absolute (*p* = 0.017) (Table 3, Fig. 3a) and relative (*p* = 0.034) weight loss, and with ferritin (Table 4, Fig. 3b) and inversely correlated with parameters of lung function, FEV1 (*p* = 0.035) (Table 3, Fig. 3c) and FVC (*p* = 0.01), cholinesterase levels (Table 5, Fig. 3d) and hemoglobin levels (Table 4, Fig. 3e). This shows a clear association of TIMP-1 plasma levels with clinical markers of cachexia.

**Table 3:** Correlations between plasma TIMP-1 and selected parameters of cachexia

		ASA	Height, m	Weight, kg	BMI, kg/m2	Weight loss, kg	Weight loss, %	Cachexia (yes or no)	Lung function: FEV1, l	Lung function: FEV1, %	Lung function: FVC, l	Lung function: FVC, %
Plasma TIMP-1, ng/ml	Spearman’s correlation	.305	−.217	.028	.161	.304	.281	.293	−.374	−.246	−.546	−.305
	Significance (2-tailed)	.027	.099	.831	.224	.017	.034	.020	.035	.175	.001	.089
	N	53	59	59	59	61	57	63	32	32	32	32

**Table 4:** Correlations between plasma TIMP-1 and selected laboratory parameters

		Plasma TIMP-1, ng/ml	Liver TIMP-1 mRNA	Pancreas TIMP-1 mRNA	CA19–9, U/ml	CEA, ng/ml	CRP, mg/dl	Hemoglobin, g/dl	Iron, μg/dl	Ferritin, ng/ml	LDH, U/l	Leucocytes, G/l	Thrombocytes, G/l	Creatinine, mg/dl
Plasma TIMP-1, ng/ml	Spearman’s correlation	1.000	.374	−.632	.074	.114	.201	−.260	−.209	.755	−.042	.119	.049	−.029
	Significance (2-tailed)	.	.188	.368	.649	.521	.246	.027	.537	.005	.864	.317	.679	.809
	N	75	14	4	40	34	35	72	11	12	19	73	73	71

**Table 5:** Correlations between plasma TIMP-1 and parameters of liver function

		Bilirubin, mg/dl	Albumin, g/dl	Protein, g/dl	Alkaline phosphatase, U/l	Cholinesterase, U/l	Quick, %	GOT, U/l	GPT, U/l	GGT, U/l
Plasma TIMP-1, ng/ml	Spearman’s correlation	.033	−.112	−.270	.228	−.414	−.098	.121	.166	.184
	Significance (2-tailed)	.785	.543	.096	.101	.010	.419	.434	.172	.124
	N	72	32	39	53	38	70	44	69	71

### Influence of cachexia and jaundice on TIMP-1 as biomarker in PDAC

**Fig. 4:**
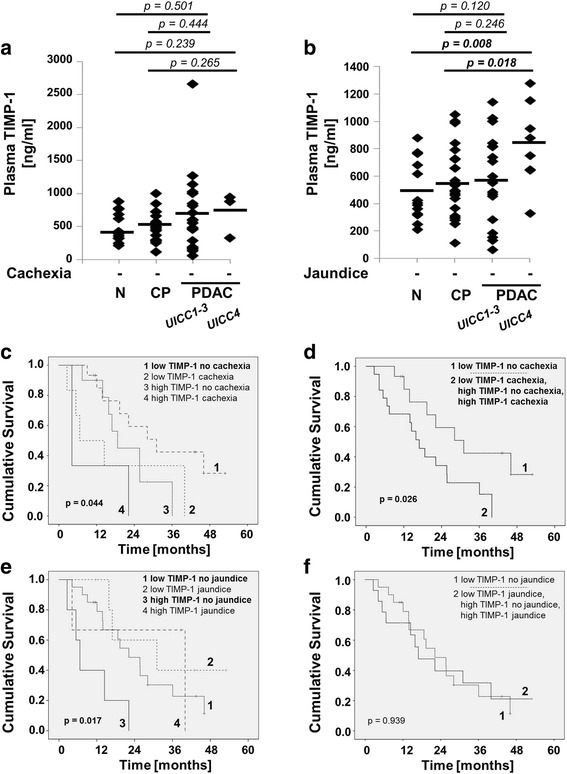
**a** Analysis of TIMP-1 in healthy individuals (control, *n* = 13), in CP (*n* = 19), PDAC patients UICC 1–3 (*n* = 20), and PDAC patients UICC 4 (*n* = 3) (patients with cachexia were excluded). Shapiro-Wilks testing in combination with visual inspection of the histograms showed that samples were not normally distributed. Groups were compared using nonparametric Mann-Whitney U test for independent variables. **b** Analysis of TIMP-1 in patients without tumor and acute inflammatory disease (control, *n* = 13), in CP (*n* = 24), PDAC patients UICC 1–3 (*n* = 18), and PDAC patients UICC 4 (*n* = 8) (patients with jaundice were excluded). Shapiro-Wilks testing in combination with visual inspection of the histograms showed that samples were normally distributed. Groups were compared using Student’s t-test for independent samples. **c**-**f** Relation of plasma TIMP-1 and PDAC-associated cachexia (**c**, **d**) and jaundice (**e**, **f**) to Kaplan Meier Overall Survival (OS) curves in PDAC patients. For the optimal cut-off at 842 ng/ml (see Fig. 1b) and presence or absence of cachexia and jaundice the Kaplan Meier curves were generated. Differences were analyzed by the Log-rank (Mantel-cox) test. **c** *p* = 0.112 low TIMP-1 no cachexia vs. low TIMP-1 cachexia; *p* = 0.097 low TIMP-1 no cachexia vs. high TIMP-1 no cachexia; ***p*** **= 0.014** low TIMP-1 no cachexia vs. high TIMP-1 cachexia; *p* = 0.978 low TIMP-1 cachexia vs. high TIMP-1 no cachexia; *p* = 0.095 low TIMP-1 cachexia vs. high TIMP-1 cachexia; *p* = 0.402 high TIMP-1 no cachexia vs. high TIMP-1 cachexia. **e** *p* = 0.322 low TIMP-1 no jaundice vs. low TIMP-1 jaundice; ***p*** **= 0.010** low TIMP-1 no jaundice vs. high TIMP-1 no jaundice; *p* = 0.670 low TIMP-1 no jaundice vs. high TIMP-1 jaundice; ***p*** **= 0.014** low TIMP-1 jaundice vs. high TIMP-1 no jaundice; *p* = 0.766 low TIMP-1 jaundice vs. high TIMP-1 jaundice; *p* = 0.152 high TIMP-1 no jaundice vs. high TIMP-1 jaundice

In the final set of analyses, we tested whether alterations in TIMP-1 plasma levels in patients with cachexia and jaundice may limit its use as a biomarker in PDAC. When patients with cachexia (Fig. 4a) or patients with jaundice (Fig. 4b) were excluded, we still observed a stepwise increase of TIMP-1 levels from healthy individuals to patients with CP and PDAC, but the differences became less drastic as compared to Fig. 1a. Interestingly, both low TIMP-1 levels and absence of cachexia were independently beneficial for survival (Fig. 4c, curve #1 vs. curve #4). Combining the two parameters (low TIMP-1 levels **and** absence of cachexia) in the survival analysis yielded an improved prognostic value (Fig. 4d curve #1) as compared to use of TIMP-1 levels alone (Fig. 1b). In contrast, jaundice interfered with the usefulness of TIMP-1 as a prognostic marker; systemic TIMP-1 levels showed a clear association with survival only when patients with jaundice were excluded (Fig. 4e curve #1 vs. curve #3). Combining both parameters diminished association of TIMP-1 levels with survival time (Fig. 4f). Thus, TIMP-1 only predicted survival in the absence of jaundice in our cohort while accounting for cachexia improved its prognostic value.

## Discussion

Our study focused on plasma TIMP-1 levels as a clinical biomarker in patients with CP and PDAC in the context of cachexia and jaundice. We report that TIMP-1 was associated with clinical markers of cachexia and also with the presence of cachexia but only in patients without jaundice. Furthermore, TIMP-1 appears to be counterindicated as a survival marker in patients with jaundice, while TIMP-1 and cachexia may be a promising combination of prognostic markers.

We are the first to report a possible association of TIMP-1 and cachexia in patients. Several laboratory (hemoglobin, ferritin, serum cholinesterase) and clinical parameters (relative and absolute weight loss, spirometry tests FEV1 and FVC) known to be associated with cachexia exhibited a significant correlation with plasma TIMP-1 levels in our study. We also found significantly higher TIMP-1 levels in patients with cachexia in the jaundice-free subgroup of our cohort. As plasma TIMP-1 levels were elevated in both, cachexia and jaundice patients due to the individual conditions, it is likely that the effects of concomitant jaundice weaken the association between TIMP-1 and cachexia. There is an urgent need to develop suitable biomarkers and potential drug targets for cancer cachexia which will facilitate better definition and earlier diagnosis of the syndrome. It is tempting to propose the use of TIMP-1 as cachexia marker, i.e. due to its involvement in muscle remodeling in cancer cachexia [26], which may allow for detection of early muscle wasting in on-setting cachexia. Future studies would benefit from increasing our patient cohort and incorporating continuous scoring methods for cachexia staging, such as CASCO score [37].

We show for the first time that TIMP-1 may be a useful prognostic marker especially in combination with cachexia but notably, not in patients with jaundice. There is a large body of evidence showing that systemic TIMP-1 levels have prognostic value in pancreatic cancer [15, 16, 21, 38]. PDAC-related conditions such as jaundice and cachexia are associated with tissue damage, which can influence TIMP-1 levels secondary to PDAC and could thus interfere with the use of TIMP-1 as a biomarker in PDAC. It was indeed reported that patients with PDAC-induced jaundice have significantly elevated levels of TIMP-1, compared to PDAC patients without jaundice, and that even benign jaundice can lead to increased TIMP-1 levels [22]. Although TIMP-1 levels were significantly raised at the time of diagnosis in cancer patients compared to healthy controls, the absolute increase observed in the absence of biliary obstruction was relatively small compared to those seen in the presence of obstruction [22]. Our results confirm this finding, demonstrating that differences in plasma TIMP-1 between PDAC, CP, and control patients are less drastic when patients with jaundice or cachexia are excluded. TIMP-1 could thus be overestimated as a diagnostic marker in case PDAC-related jaundice and cachexia are not accounted for. This is by the observation that jaundice and cachexia patients had higher plasma TIMP-1 level. Interestingly, this increase in TIMP-1 levels was similar in both conditions but had clearly different consequences for the use of TIMP-1 as prognostic factor: Presence of jaundice clearly interfered with the prognostic value of TIMP-1, and the impact of TIMP-1 on survival became most evident when jaundice patients were excluded. We thus emphasize that it is essential to consider whether or not a patient has jaundice when using TIMP-1 as a biomarker. In fact, this limitation is not restricted to TIMP-1 and CA19–9, the most established tumor marker for PDAC with a reported sensitivity of 79% and specificity of 82% [39], was shown to be influenced by jaundice [40]. There is also a positive correlation between bilirubin and CA19–9 in benign jaundice whereas no such relationship exists for malignant jaundice cases [40]. In contrast, the higher TIMP-1 levels of cachexia patients did not interfere with usefulness of TIMP-1 as prognostic marker and excluding cachectic patients even reduced the association of TIMP-1 with survival. Moreover, combining TIMP-1 plasma levels with cachexia status improved the prognostic value, suggesting this newly identified association between TIMP-1 and cachexia could provide a benefit for patient stratification.

## Conclusions

We show that TIMP-1 was counterindicated as a marker in patients with jaundice, while TIMP-1 together with cachexia appeared as a promising combination of prognostic parameters. We report for the first time that TIMP-1 was associated with presence of cachexia and cachexia-associated clinical markers, and conclude that TIMP-1 should be further evaluated as a cachexia biomarker. We emphasize that careful clinical evaluation of the patient, under consideration of PDAC-related secondary conditions, must be recognized as the basis for a meaningful interpretation of molecular biomarkers.

## Abbreviations

BMI: Body mass index
CRP: c-reactive protein
CT: Computed tomography
FEV_1_: Forced expiratory volume at the end of the first second of forced expiration
FVC: Forced vital capacity
PDAC: Pancreatic ductal adenocarcinoma
TIMP-1: Tissue inhibitor of metalloproteinases-1
UICC: the International Union against Cancer
